# Crucial role of the MAPKAP kinases 2 and 3 for pathogen-induced inflammation and their relevance for the immune response of the liver

**DOI:** 10.1186/2047-783X-19-S1-S24

**Published:** 2014-06-19

**Authors:** Christian Ehlting, Dieter Häussinger, Johannes G Bode

**Affiliations:** 1 Clinic of Gastroenterology, Hepatology and Infectious Diseases, Heinrich Heine University, 40225 Düsseldorf, Germany

## 

The liver plays an important role in innate and adaptive immunity and in particular in induction of tolerance. The liver is frequently exposed to pathogens like gut-derived food antigens, environmental toxins and bacterial or viral products reaching the liver via the blood flow. It is central for production of acute phase proteins, among others including protease inhibitors, soluble pattern-recognition receptors and components of the complement system, which are important constituents of innate immunity. The liver harbors the largest pool of sessile tissue macrophages within the body, mainly localized within the periportal area of the sinusoids at a strategically important position. These resident tissue macrophages play a key role for the elimination of opsonized immune complexes by phagocytosis and are important sources of inflammatory cytokines, which induce acute phase protein production in hepatocytes and further trigger innate immunity and subsequent formation of adaptive immunity. Within macrophages the MAPKAP kinases (MK)2 and 3, which represent downstream targets of the MAP kinase family member p38^MAPK^, are known to own key functions in the coordination of the inflammatory response. Thereby, their role for the regulation of the expression of inflammatory as well as anti-inflammatory cytokines, such as TNF-α, IL-1β, IL-6, IFN-γ and IL-10 has been mainly investigated in the context of bacterial components and in particular in the context of lipopolysaccharide (LPS). In this context MK2 and MK3 have been suggested to act in a co-operative manner as the expression of these cytokines is abrogated upon deletion of MK2 and is further diminished by the additional deletion of MK3. However, while investigating the role of MK2 and MK3 for the regulation of LPS-induced IFN-β production our group recently provided evidence that MK2 and MK3 are also able to exert rather distinct than co-operative regulatory effects on gene expression [1]. At the current stage the data suggest that these distinct regulatory effects of MK2 and MK3 become apparent, if regulation of respective target gene expression by these two kinases exclusively occurs at the level of transcription and does not involve post-transcriptional regulatory mechanisms such as regulation of transcript stability. Contrariwise, if regulation of gene expression by MK2 and MK3 occurs at the level of transcript stability these two kinases mainly seem to act co-operatively. Thus, MK2 and to a lesser extent MK3 are critical for regulation of TNF-α, IL-6 and IL-10 in response to LPS, where they are involved in control of transcript stability or translation. This is in contrast to the regulation of IFN-β gene expression by MK2 and MK3 where MK2 controls IFN-β gene expression by neutralizing inhibitory effects of MK3, which in the absence of MK2 inhibits transcriptional activation of IFN-β gene expression by impeding IRF-3 protein expression as well as LPS-induced nuclear translocation of NFκB [1]. Of note, unlike for example regulation of LPS-induced IL-10 expression, which essentially requires MK2 for stabilization of the IL-10 transcript, the stability of the IFN-β transcript does not require the presence of MK2.

Extended analysis of LPS-induced gene expression in macrophages derived from wild-type animals or from animals deficient for MK2 or MK2 and MK3 using “whole genome microarrays” suggested that there is a larger group of genes, which are controlled by MK2 and MK3 in a way that is comparable to that of IFN-β. Interestingly, these studies further revealed that the deletion of MK2 or of both MK2 and MK3 results in massive alterations of the inflammatory response of macrophages towards LPS, which also includes that approximately 30% of genes are only regulated by LPS if MK2 or MK2 and MK3 are absent. This indicates that apart from being critically involved in positive or negative regulation of gene expression in response to LPS MK2 and MK3 are also required to prevent a larger group of genes from being regulated in response to LPS.

The role of MK2 is comparatively well studied in the context of bacterial infections and the inflammatory host response towards bacteria-derived pathogens such as LPS [2] whereas its role for viral infections and virus-host interactions is less well characterized. Apart from other inflammatory mediators cytomegalovirus (CMV) induces the expression of the anti-inflammatory cytokine IL-10 that dampens activation of Th1 cells, NK cells and macrophages, which are required for optimal pathogen clearance, but also contribute to tissue damage during infection. Furthermore the expression of IL-10 limits immune cell activation and suppresses IFN-γ-induced MHC class I and II surface expression, thereby escaping anti-viral mechanisms and diminishing the pro-inflammatory response. This is of particular importance for the prevention of CMV-induced liver pathogenesis as suggested from studies on IL-10-deficient mice, which upon infection with murine (M)CMV develop an exaggerated pro-inflammatory cytokine response and enhanced liver injury, characterized by the increased induction of apoptotic cell death and enlarged cellular infiltration. However, the molecular mechanisms involved in CMV-induced IL-10 expression are unclear.

Our studies reported herein propose a relevance of MK2 for MCMV-infection *in vivo* and characterizes its role for virus-induced IL-10 expression *in vitro* and *in vivo*. The data presented indicate that in macrophages MK2 is crucial for MCMV-induced IFN-β expression, which in turn represents an important mediator for sustained IL-10 production in response to MCMV infection as suggested from studies using macrophages isolated from interferon alpha receptor (IFNAR)1-deficient mice or wild-type macrophages treated with antagonizing antibodies specific for IFNAR1. These data suggest that type I IFN driven feedback loops are critical for virus-induced production of IL-10. In addition, MK2 is further essential for the stabilization of the IL-10 transcript. Thereby, MK2 stabilizes the IL-10 transcript most likely by preventing destabilizing effects of tristetraproline (TTP), which is expressed in the host cell upon MCMV-infection to exert a negative feedback regulation on IL-10 expression. Apart from IL-10 and IFN-β MK2 is further demonstrated to be necessary for MCMV-induced production of other cytokines such as IL-6 and TNF-α, whereas it is dispensable for the production of IFN-γ. As a consequence this results in an increased IFN-γ/IL-10 ratio, which may be responsible for the enhancement of MHC class I and II expression observed in the liver tissue of MCMV-infected MK2-deficient mice. Enhanced expression of MHC proteins in turn may be involved in the observed formation of intrahepatic CD11b+ and CD8+ mononuclear cell aggregates and in an enhancement of apoptotic cell death observable in the liver tissue of MCMV-infected MK2-deficient mice. The latter may contribute to the fact that despite of profound alterations of cytokine expression, including impaired expression of IFN-β, IL-6, IL-10 and TNF-α in response to MCMV infection, viral clearance is not impaired in MK2-deficient mice.
